# Nanobiotechnological Nanocapsules Containing Polyhemoglobin-Tyrosinase: Effects on Murine B16F10 Melanoma Cell Proliferation and Attachment

**DOI:** 10.1155/2012/673291

**Published:** 2012-11-08

**Authors:** Yun Wang, Thomas M. S. Chang

**Affiliations:** Artificial Cells and Organs Research Centre, Departments of Physiology, Medicine and Biomedical Engineering, Faulty of Medicine, McGill University, Room 1004, 3655 Drummond Street, Montreal, QC, Canada H3G 1Y6

## Abstract

We have reported previously that daily intravenous infusions of a soluble nanobiotechnological complex, polyhemoglobin-tyrosinase [polyHb-Tyr], can suppress the growth of murine B16F10 melanoma in a mouse model. In order to avoid the need for daily intravenous injections, we have now extended this further as follows. We have prepared two types of biodegradable nanocapsules containing [polyHb-Tyr]. One type is to increase the circulation time and decrease the frequency of injection and is based on polyethyleneglycol-polylactic acid (PEG-PLA) nanocapsules containing [polyHb-Tyr]. The other type is to allow for intratumoural or local injection and is based on polylactic acid (PLA) nanocapsules containing [polyHb-Tyr]. Cell culture studies show that it can inhibit the proliferation of murine B16F10 melanoma cells in the “proliferation model”. It can also inhibit the attachment of murine B16F10 melanoma cells in the “attachment model.” This could be due to the action of tyrosinase on the depletion of tyrosine or the toxic effect of tyrosine metabolites. The other component, polyhemoglobin (polyHb), plays a smaller role in nanocapsules containing [polyHb-Tyr], and this is most likely by its depletion of nitric oxide needed for melanoma cell growth.

## 1. Introduction

Deregulated proliferation and differentiation of melanocytes lead to the formation of melanoma [1]. Although not as common as the other skin basal cell skin cancer or skin squamous cell cancer, melanoma is far more dangerous. Surgical removal is effective in the early stage. However, once it has metastasized beyond the local lymph nodes, it is eventually fatal [2–4]. Chemotherapy, radiotherapy, and other approaches in combination are being investigated [5–10].

 Melanoma cells show specific amino acid-dependence for Tyrosine (Tyr) and phenylalanine (Phe) [11–14] and also arginine [15, 16]. Tyr/Phe deprivation induces G_0_/G_1_ cell cycle arrest in murine melanoma [17] and induces apoptosis by inhibiting integrin/focal adhesion kinase (FAK) pathway and activating caspases [18–20]. Tyr/Phe deprivation induces apoptosis in murine and human melanoma cells but not in normal cells [21]. One method is to utilize the tyrosinase-dependent catalytic reaction to suppress Tyr level and also consume Phe [22, 23]. In addition, the generated products of tyrosine metabolism, such as dopa, 5,6-dihydroxyindole (DHI), 5,6-dihydroxyindole-2-carboxylic acid (DHICA) and others, are also toxic to melanoma cells [24]. Oxidation of these tyrosine metabolites can produce reactive oxygen species [24]. In the melanoma cells, the excessive reactive oxygen species will stimulate cell apoptosis by activating DNA damage-repair pathway and also opening mitochondrial pore [25]. Despite the potential of tyrosinase, injection of free enzymes has problems related to immunology, stability, and duration of action.

 Artificial cells bioencapsulated enzymes were first prepared for different medical applications [26]. This approach has shown potentials in catalase for the depletion of hydrogen peroxide in acatalasemia [27], asparaginase for the depletion of asparagine for 6C3HED lymphosarcoma [28], phenylalanine ammonia-lyase for the depletion of phenylalanine in phenylketonuria [29], and xanthine oxidase to remove hypoxanthine in Lesch-Nyhan Disease [30]. Detailed *in vitro* studies show that the enzyme in artificial cells no longer has immunological problems [31]. Two further developments have led to the possible clinical applications of these animal studies. One is the development of nanobiotechnological approach for artificial cells [32]. Another development is the first artificial cells with biodegradable polymeric membrane [33]. These have now led to extensive developments in this area [34–36].

 One area is the clinical applications of oxygen therapeutics using the basic nanobiotechnological procedure of crosslinking hemoglobin [32] to form soluble polyHb [37, 38]. PolyHb has been tested in phase III clinical trials as blood substitutes [39, 40] and has now been approved for routine clinical patient uses in Russia and South Africa. These oxygen carriers have also been tested in animal studies and found to increase tissue oxygenation and enhance radiation and chemotherapy in solid tumors [41, 42]. We have methods for the crosslinking of enzymes to hemoglobin to form polyHb-enzyme systems [37, 38, 43]. We therefore studied the crosslinking of tyrosinase to hemoglobin to form polyHb-tyrosinase [44, 45]. The increased tissue oxygenation could then be an additive effect on Tyr depletion for melanoma. Our studies show that this approach can significantly suppress the growth of murine B16F10 melanoma mice [44]. However, polyHb-tyrosinase requires daily intravenous infusions. Furthermore, polyHb-tyrosinase is a solution that does not stay at the site of intratumoural and local injection. This solution also cannot be located at the drainage lymphatic nodes or organs. We are in the process of improving this approach by combining the nanobiotechnological approach of polyHb-tyrosinase with biodegradable nanodimension artificial cells. We have developed the original biodegradable polymeric artificial cells [33] into a nanodimension system that can be given intravenously [46]. PolyHb in PEG-PLA membrane nanodimension artificial cells has a much longer circulation time than polyHb [47]. Infusion of 1/3 the total blood volume into rats does not cause long-term adverse effects on the histology and function of liver, spleen, and kidney [48, 49]. Other centers are now using this approach [50–53]. 

 In order to avoid the need for daily intravenous injections, in the present study we have prepared two types of biodegradable nanocapsules containing [polyHb-Tyr]. One type is to increase the circulation time and decrease the frequency of injection and is based on PEG-PLA nanocapsules containing [polyHb-Tyr]. The other type is to allow for intratumoural or local injection and is based on PLA nanocapsules containing [polyHb-Tyr]. The cell culture study shows that it can inhibit the proliferation of murine B16F10 melanoma cells in the “proliferation model.” It can also inhibit the attachment of murine B16F10 melanoma cells in the “attachment model.” The component analysis shows that the enzymatic action of tyrosinase on tyrosine plays the major role in nanocapsule-[polyHb-Tyr]. This could be due to the depletion of tyrosine or the toxic effects of the metabolites of tyrosine. The other component, polyHb, plays a smaller role in nanocapsule-[polyHb-Tyr], and this is most likely due to its depletion of nitric oxide needed for melanoma cell growth. 

## 2. Materials and Methods

### 2.1. PolyHb and PolyHb-Tyrosinase Preparations

This is as described in detail elsewhere [36, 44, 45]. Briefly, stroma-free hemoglobin (SFHb) with a concentration of 7 g/dL Hb with or without tyrosinase was dissolved in a 0.1 mol/L sodium phosphate buffer (pH = 7.4). Lysine was added at a molar ratio of 7 : 1 lysine/hemoglobin. Glutaraldehyde as a crosslinker was added at a molar ratio of 16 : 1 glutaraldehyde/hemoglobin. The process of crosslink continued for 24 hours. After that, lysine was added again to stop the crosslink reaction at a molar ratio of 200 : 1 lysine/hemoglobin. The sample was dialyzed overnight with a dialysis membrane (molecular weight cut-off = 12–14 kDa) and then passed through sterile 0.45 *μ*m syringe filters to remove impurities. All operations were conducted at 4°C and under nitrogen in order to prevent the formation of methemoglobin and the degradation of enzyme.

### 2.2. Tyrosinase Nanocapsules Preparation

**Figure 1:**
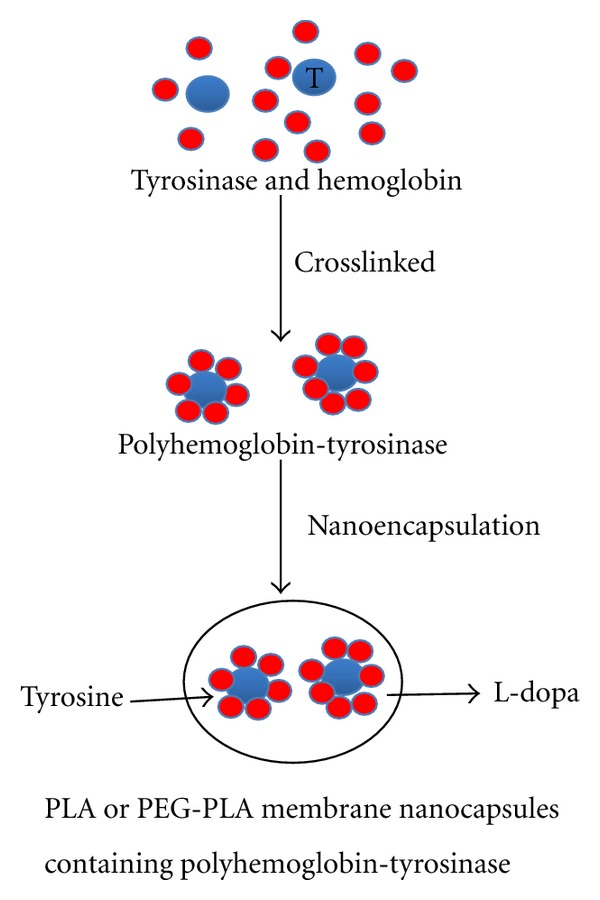
Schematic representation of the preparation of nanobiotechnological procedure for the PLA or PEG-PLA membrane nanocapsules containing polyhemoglobin-tyrosinase. Tyrosinase: larger blue circles. Hemoglobin: smaller red circles.

Nanocapsules were prepared as described [54] (Figure 1). Briefly, 50 mg PLA and 25 mg hydrogenated soybean phosphatidylcholine were firstly dissolved in 4 mL acetone and 2 mL ethanol by sonication for 30 min. The formed organic phase was dropwise added into 5 mL 7 g/dL polyHb-Tyr solution with a syringe under moderate magnetic stirring. Then the suspension was stirred continually for 30 min. The organic solvent was removed using rotary evaporator under vacuum for about 1 hour. Then the resultant nanocapsules were separated by centrifugation at 20,000 rpm for 20 min to separate the supernatant and sediment. Then the suspension was concentrated with filtration. 

### 2.3. Molecular Weight Distribution

Sephacryl-300 HR column (*V* total = 560 mL) was used for molecular weight distribution analysis of polyHb-Tyr. The elution buffer (0.1 M Tris-HCl and 0.15 M NaCl (pH 7.4) at a flow rate of 36 mL/hour) was monitored by a 280 nm UV detector. Three molecular weight fractions were collected (1) >450 kDa, (2) 100–450 kDa, and (3) >100 kDa.

### 2.4. Tyrosinase Activity Assay

In our assay, tyrosinase catalyzes the substrate tyrosine into L-dopa under O_2_, and the formed L-dopa is then converted into L-dopa-quinone and H_2_O. Tyrosinase activity was tracked by monitoring the production rate of the enzymatic product L-dopaquinone at 300 nm, as described previously [54]. Briefly, the reaction solution was prepared with 0.34 mM L-tyrosine and 12.8 mM potassium phosphate buffer. This solution was adjusted to pH 6.5 and oxygenated by bubbling oxygen through for 10 minutes before usage. 10 to 100 *μ*L of samples were mixed with the reaction solution 2.9 mL in a cuvette. Absorbance at 300 nm was monitored. The generated absorbance values were converted into enzyme activity unit by comparing with standard curves.

### 2.5. Tyrosinase Entrapment Efficiency

Drug entrapment efficiency of the nanocapsule was calculated using the following equation:

(1) $$\begin{array}{c} \text{Entrapment}\;\text{efficiency}\;\left(\%,\text{w}/\text{w}\right)=\frac{\text{Tyr}\;\text{activity}\;\text{in}\;\text{polyHb-Tyr}-\text{Tyr}\;\text{activity}\;\text{in}\;\text{nanocapsules'}\;\text{supernatant}}{\text{Tyr}\;\text{activity}\;\text{in}\;\text{the}\;\text{polyHb-Tyr}}. \end{array}$$

### 2.6. Physicochemical Characterization of Nanocapsules

The size and morphological examination of nanocapsules were performed by Transmission electron microscopy (TEM). Specifically, about 10 uL nanocapsule samples were placed on 200 mesh carbon-coated copper grids and then examined with a JEOL JEM-2000FX microscope (Jeol Ltd., Tokyo, Japan) and photographed with a Gatan Wide Angle Multiscan CCD Camera.

### 2.7. Tumour Cells and Culture Conditions

B16F10 murine melanoma cells (American Type Culture Collection, ATCC, #CRL-6475) were cultured in standard Dulbecco's modified Eagle's medium (DMEM) supplemented with 10% fetal bovine serum at 37°C and 5% CO_2_, humidified atmosphere. Cells were passaged every 2-3 days. When the cells reached 90–100% confluence, they were washed with 0.25% trypsin/EDTA for about 1 min to be detached from the dish. Then cell suspensions were collected in a sterile 15 mL tube and centrifuged at 1,500 rpm for 5 min. Cells were counted by trypan blue exclusion and suspended in fresh medium to the desire density.

### 2.8. Cell Proliferation Assays

5 × 10^4^ Melanoma cells in 2 mL complete DMEM were seeded in 6-well plates. When the tumor cells became 30–40% confluent, different nanocapsules or the same amount of saline were mixed with DMEM and added into each well. Cells were cultured for 0, 24, and 48 hours and collected by 0.25% trypsin/EDTA. The cell proliferative ability was then determined by trypan blue exclusion. The data from triplicate wells were averaged, and each experiment was repeated three times. 

### 2.9. Cell Attachment Assays

For the experiment, melanoma cells suspension was mixed with different nanocapsules formats or the same amount of saline in the complete DMEM. Then the cells together with nanocapsules were mixed well and seeded in 6-well plates for 0, 24, and 48 hours observation. Use PBS to wash the unattached tumor cells, and collect the attached tumor cells by 0.25% trypsin/EDTA. The cell attachment ability was then determined by trypan blue exclusion. The data from triplicate wells were averaged, and each experiment was repeated three times. 

### 2.10. Data Analysis

Statistical analysis was performed using the Student's *t*-test within analysis of variance and was considered to be significant at *P* < 0.05.

## 3. Results

### 3.1. Physicochemical Characteristics of PLA Nanocapsule [polyHb-Tyr]

**Figure 2:**
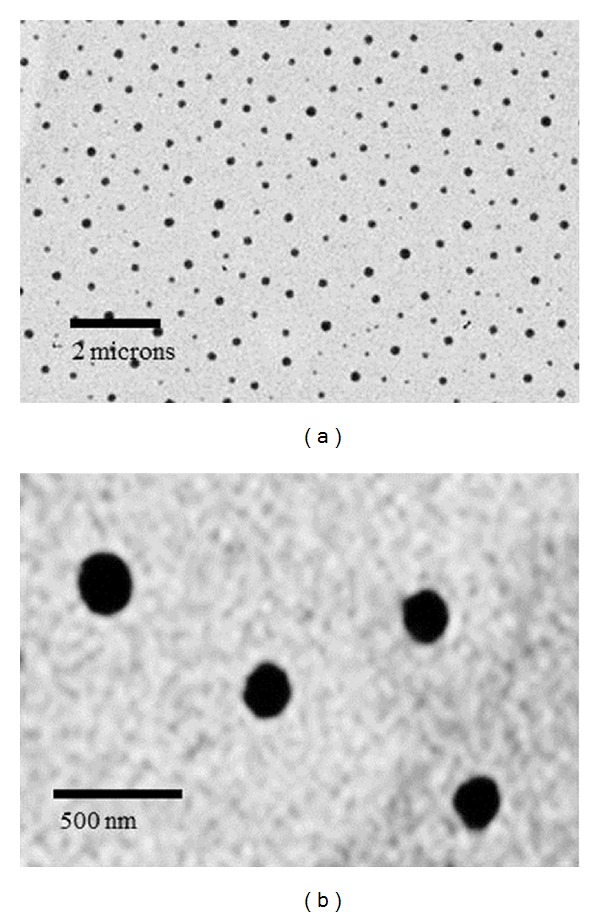
Physicochemical characteristics of PLA nanocapsule [polyHb-Tyr]. (a) TEM of PLA nanocapsule [polyHb-Tyr] at low magnification. (b) TEM of PLA nanocapsule [polyHb-Tyr] at high magnification.

**Figure 3:**
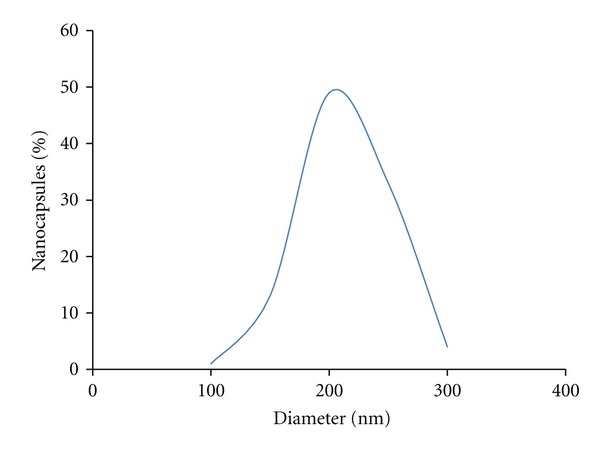
Size distribution of PLA nanocapsule [polyHb-Tyr].

TEM image showed that the PLA nanocapsule [polyHb-Tyr] was spherical with smooth surface and freely dispersed (Figures 2(a) and 2(b)). The size distribution based on the TEM images showed a range from 88 to 267 nm (187 ± 35 nm), and 64% of the nanocapsules are in the range of 120 to 200 nm (Figure 3).

### 3.2. Nanoencapsulation Efficiency of PLA Nanocapsule [polyHb-Tyr]

**Figure 4:**
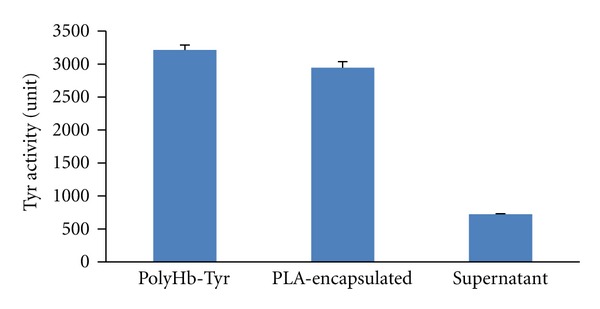
Entrapment efficiency of PLA nanocapsule [polyHb-Tyr].

Sepacryl S-300 gel column chromatography was used to analyze the molecular weight distribution of polyHb-Tyr. The molecular weight distributions for polyHb-Tyr showed three types of molecular weight distributions: (1) low (<100 kDa), (2) intermediate (100–450 kDa), and (3) high molecular weight (>450 kDa). The low molecular weight fraction (<100 kDa) (11.5% of the sample) was discarded. The fraction of >100 kDa (88.5% of the samples) was used for nanoencapsulation. The encapsulation efficiency was 75.4% (Figure 4).

### 3.3. Effects of PLA Nanocapsule [polyHb-Tyr] on the Proliferation of B16F10 Cells

**Figure 5:**
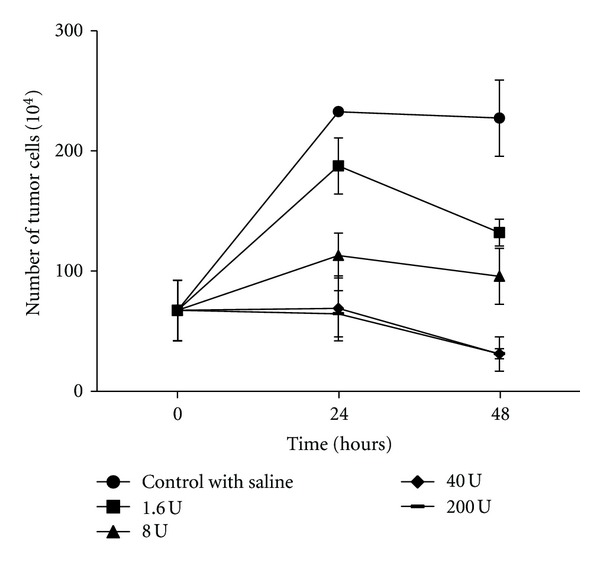
The effects of different amounts of PLA nanocapsule [polyHb-Tyr] on the proliferation of B16F10 melanoma cells.

**Figure 6:**
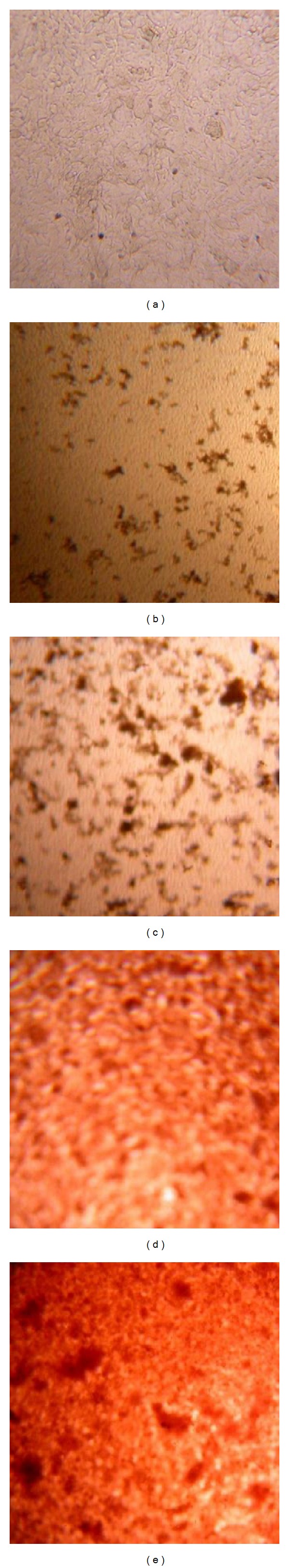
Effects of PLA nanocapsule [polyHb-Tyr] on the proliferation of B16F10 melanoma cells. The microscopy images of B16F10 melanoma cells treated with the nanocapsules containing enzyme activities increasing in enzyme activity from (a) to (e): 0, 1, 6, 8, 40, 200 units.

We treated B16F10 melanoma cells with PLA nanocapsules [polyHb-Tyr] containing a large range of enzyme activities, from 1.6 units to 200 units. The result showed that PLA nanocapsule [polyHb-Tyr] inhibited tumor proliferation, and the effect was tyrosinase dose dependence. Compared with the control group, the higher the tyrosinase activity the greater was the inhibition of tumour proliferation as shown by the decrease in cell viability at 24 hours and 48 hours. At 48 hours, the tumour cells growth was most effectively inhibited in the groups of 40 units and 200 units of tyrosinase activity (Figure 5). The control group showed normal morphology with spindle-shaped and epithelial-like features (Figure 6(a)); after the treatment with PLA nanocapsule [polyHb-Tyr], dead cell clusters were observed with more dead cell clusters in the higher tyrosinase groups (Figures 6(b) to 6(e)).

**Figure 7:**
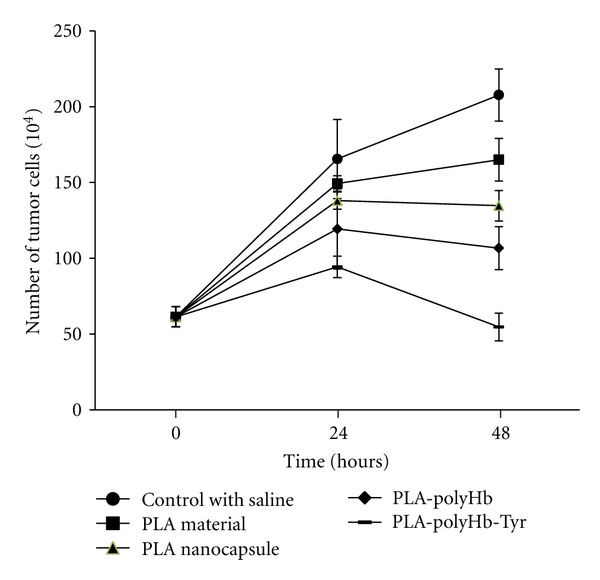
PLA nanocapsule [polyHb-Tyr] and the effects of its components on the proliferation of B16F10 melanoma cells.

 We also carried out a study to analyze the different PLA nanocapsule [polyHb-Tyr] components on the inhibition of cells proliferation (Figure 7). The components tested included the PLA nanocapsule [polyHb-Tyr] group, the PLA material, empty PLA nanocapsules, and PLA nanocapsule [polyHb] without tyrosinase. The result showed that PLA nanocapsule [polyHb-Tyr] was responsible for the major role in the inhibition of cells proliferation. PLA nanocapsule [polyHb] can also suppress the tumor cells growth although not as much as that of the PLA nanocapsule [polyHb-Tyr]. In addition, the PLA material and empty PLA nanocapsule showed a very slight activity. Collectively, these results suggested that the PLA nanocapsule containing polyHb-Tyr had the ability to inhibit the tumor cells proliferation and the tyrosinase contributed to the major effect.

### 3.4. Effects of PLA Nanocapsule PolyHb-Tyr on the Attachment of B16F10 Cells

**Figure 8:**
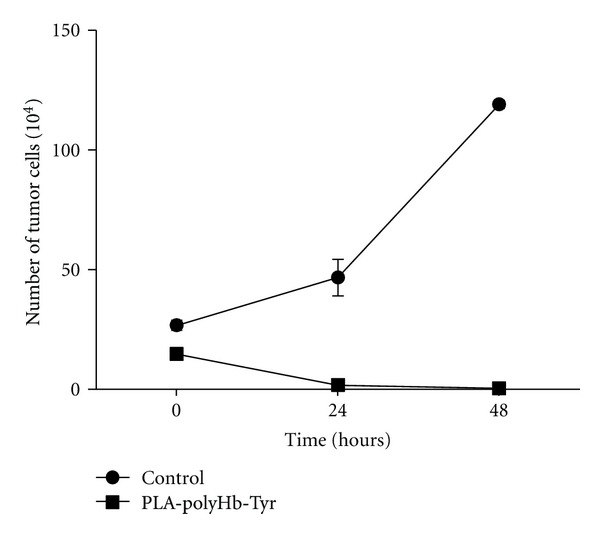
The effects of PLA nanocapsule [polyHb-Tyr] on the attachment ability of B16F10 melanoma cells.

For the solid cancer melanoma, the metastatic dissemination from a primary lesion to a secondary site is believed to be the major reason leading to death [55, 56]. The tumor cells attached to the secondary site is one of the vital steps of this metastatic process [57]. Thus, we carried out the following experiment to identify the effect of PLA nanocapsule [polyHb-Tyr] on melanoma cell attachment. We mixed melanoma cells and PLA nanocapsule [polyHb-Tyr] and cocultured the suspension solution in 6 well plates to observe the cell viability. It was found PLA nanocapsule [polyHb-Tyr] effectively inhibited tumor cells attachment to plastic plates. In 24 hours, most of the tumor cells died and by 48 hours of culture there were no viable cells (Figure 8).

**Figure 9:**
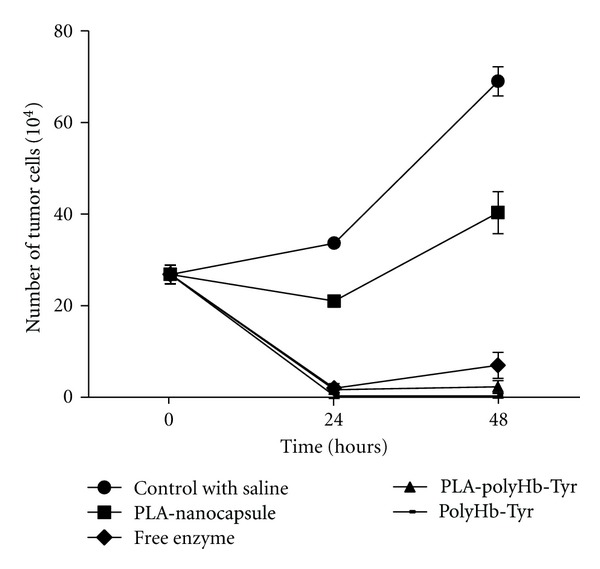
The effects of PLA nanocapsule [polyHb-tyr] and the functions of its components on the attachment of B16F10 melanoma cells.

 We also tested the different components of PLA nanocapsule [polyHb-Tyr]. Saline was used as negative control and the free tyrosinase as positive control. Free tyrosinase reached its maximal effect at 24 hours. PolyHb-Tyr could inhibit tumor cells attachment in 24 hours culture and maintain this function to 48 hours. At 24 hour, PLA nanocapsule [polyHb-Tyr] was as effective as free enzyme and polyHb-Tyr and this was maintained for 48 hours. Empty PLA nanocapsules had a slight effect at 24 hours, but this effect was minimal by 48 hours (Figure 9).

**Figure 10:**
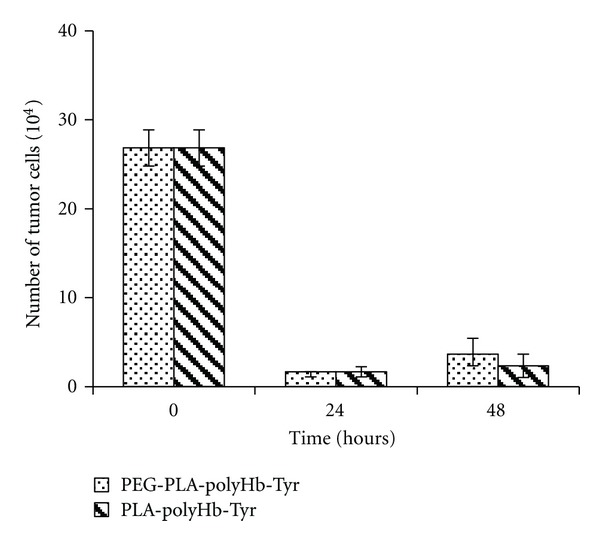
The functions of PEG-PLA and PLA nanocapsules on the attachment of B16F10 melanoma cells.

 PLA nanocapsule [polyHb-Tyr] is for the use in intratumoural and local injection. For intravenous injection, we have prepared PEG-PLA nanocapsule [polyHb-Tyr]. A comparison of PEG-PLA nanocapsule [polyHb-Tyr] and PLA-nanocapsule [polyHb-Tyr] showed that there were no significant differences in their effects on B16F10 melanoma cells (Figure 10). 

## 4. Discussion

Oxygen carriers increase tissue oxygenation in poorly perfused solid tumours and increase the sensitivity of the tumours to radiation and chemotherapy [41, 42]. By crosslinking tyrosinase to hemoglobin to form polyhemoglobin-tyrosinase, we can simultaneously act on tyrosine and increase oxygenation for melanoma cells to be more sensitive to the lowered tyrosine level or the toxic effects of tyrosine metabolites, resulting in the inhibition of murine B16F10 melanoma growth in mice [44, 45]. To avoid the need for daily intravenous infusions, we have prepared PEG-PLA nanocapsules containing the nanobiotechnological complex of polyhemoglobin-tyrosinase [54]. This has a much longer circulation time than the polyhemoglobin complex [47]. By using PLA instead of PEG-PLA, we can prepare PLA nanocapsule [polyHb-Tyr] for intratumoral or local injection around the tumour sites or for locating at the lymph nodes that drain the area. The smaller nanocapsules can enter the melanoma cells to inhibit cell growth and attachment especially if the cells metastasize. The larger ones can act on tyrosine in the extracellular environment of the melanoma cells to convert extracellular tyrosine. They can also follow the same path as the metastasis of melanoma cells to the lymph nodes or organs to continue to act on tyrosine in the region. The diameters are in the range of 88 to 267 nm (187 ± 35 nm). This would have nanocapsules for both intracellular and extracellular functions. A range of 33 to 300 nm in diameter is also possible. 

 At present, two types of experiments on murine B16F10 melanoma cells were carried out. (1) Proliferation model: attached B16F10 melanoma cells were treated with PLA nanocapsules containing polyHb-Tyr or the different components. The effects on cell viability were followed. (2) Attachment model: melanoma cells were cocultured with PLA nanocapsules containing polyHb-Tyr or the different components. The effects on cell attachment were followed. The data showed that PLA nanocapsule [polyHb-Tyr] could inhibit both the proliferation and attachment of melanoma cells. This is related to the enzymatic action of tyrosinase on tyrosine. This could be due to the lowered tyrosine level [17–20] or the toxic effect of products tyrosine metabolism [24, 25]. The results also showed that polyhemoglobin, one of the components of PLA nanocapsule [polyHb-Tyr], had by itself some ability to inhibit melanoma cells growth. Hemoglobin is a known nitric oxide scavenger [58] and the depletion of endogenously produced nitric oxide can inhibit melanoma proliferation and promote apoptosis [59]. As a result, polyhemoglobin in the PLA nanocapsule [polyHb-Tyr] could have an additional effect on limiting the growth of melanoma cells. For intravenous use, PEG is added to PLA to form PEG-PLA nanocapsule [polyHb-Tyr] resulting in much longer circulation time. The present study showed that it was also as effective as PLA nanocapsule [polyHb-Tyr] in inhibiting melanoma cells attachment. 

## 5. Conclusion

Previously, we have reported that daily intravenous infusions of a soluble nanobiotechnological complex, polyhemoglobin-tyrosinase [polyHb-Tyr], can suppress the growth of a murine B16F10 melanoma in a mouse model [44]. In order to avoid the need for daily intravenous injections, we have now prepared two types of biodegradable nanocapsules containing [polyHb-Tyr] with diameters in the range of 88 to 267 nanometers. One type is to increase the circulation time and decrease the frequency of injection and is based on PEG-PLA nanocapsules containing [polyHb-Tyr]. The other type is to allow for intratumoural or local injection and is based on PLA nanocapsules containing [polyHb-Tyr]. The cell culture study shows that it can inhibit the proliferation of murine B16F10 melanoma cells in the “proliferation model.” It can also inhibit the attachment of murine B16F10 melanoma cells in the “attachment model.” The component analysis shows that the enzymatic action of tyrosinase on tyrosine plays the major role in the nanocapsule-[polyHb-Tyr]. The other component, polyHb, plays a smaller role in the nanocapsules containing [polyHb-Tyr], and this is most likely due to its depletion of nitric oxide needed for melanoma cell growth.
